# Contextual and mental health service factors in mental disorder-based disability pensioning in Finland – a regional comparison

**DOI:** 10.1186/s12913-021-07099-4

**Published:** 2021-10-11

**Authors:** Tino Karolaakso, Reija Autio, Turkka Näppilä, Helena Leppänen, Päivi Rissanen, Martti T. Tuomisto, Sakari Karvonen, Sami Pirkola

**Affiliations:** 1 Faculty of Social Sciences (Psychology), Tampere University, Arvo Ylpön katu 34, FI-33520 Tampere, Finland; 2 Faculty of Social Sciences (Unit of Health Sciences), Tampere University, Tampere, Finland; 3 Tampere University Library, Tampere University, Tampere, Finland; 4 Faculty of Medicine and Health Technology, Tampere University, Tampere, Finland; 5 Public Health and Welfare Division, Finnish Institute for Health and Welfare, Helsinki, Finland; 6 Department of Adult Psychiatry, Tampere University Hospital, Pirkanmaa Hospital District, Tampere, Finland

**Keywords:** Mental disorders, Disability pension, Regional differences, Contextual factor, Compositional factor, Mental health services

## Abstract

**Background:**

We investigated the regional differences in all mental disorder disability pensions (DP) between 2010 and 2015 in Finland, and separately in mood disorders and non-affective psychotic disorder DP. We also studied the contribution of several district-level contextual and mental health service factors to mental disorder DP.

**Methods:**

Subjects were all those granted mental disorder DP for the first time between 2010 and 2015 in Finland (*N* = 36,879). Associations between the district-level contextual and mental health service factors and regional DP risks collected from the year 2015 were studied with negative binomial regression analysis in the Finnish hospital districts. The population number on the age (18 to 65 years), gender, occupational status and residential hospital district of the Finnish population from 2015 was used as exposure in the model.

**Results:**

Significant differences in the regional mental disorder DP risks between and within hospital districts did not appear to follow the traditional Finnish health differences. A lower risk of DP was associated with contextual indicators of higher regional socioeconomic level. Furthermore, population density as a proxy for access to mental health services indicated a higher regional DP risk for lower density in all mental (IRR 1.10; 95% CI 1.06–1.14) and mood disorder (IRR 1.12; 95% CI 1.08–1.16) DP. Both the highest and the lowest regional numbers of all mental health outpatient visits were associated with a higher DP risk in all mental and mood disorder DP, whereas particularly low regional numbers of inpatient treatment periods and of patients were associated with a lower risk of DP.

**Conclusions:**

In this comprehensive population-level study, we found evidence of significant regional variation in mental disorder DP and related district-level factors. This variation may at least partly relate to differences in regional mental health service systems and treatment practices. Adapting to the needs of the local population appears to be indicated for both regional mental health service systems and treatment practices to achieve optimal performance.

**Supplementary Information:**

The online version contains supplementary material available at 10.1186/s12913-021-07099-4.

## Background

It is estimated that in the EU, approximately 38% of the adult population is affected every year by at least one mental disorder. There is little evidence of significant inter-country variation [1]. The economic burden of mental health problems is considerable. Total costs in EU are estimated by the OECD at around 4% of gross domestic product (GDP), meaning more than 600 billion euros annually [2]. One economically and humanely most expensive mental disorder outcome is loss of one’s working ability, resulting in a disability pension (DP).

Significant regional differences in many Western countries have been reported in overall DP [3–9] and in mental disorder-related sickness absence and DP [10–14]. Factors contributing to the regional differences in health and DP can be categorized to 1) individual-level compositional factors, for example age- and socioeconomic status distribution of the region’s population, 2) district-level contextual sociodemographic and socioeconomic factors, such as minority- and employment rates and economic prosperity, and 3) district-level health care service factors, for example number of outpatient visits and patients in inpatient care [15, 16];. Previous research has suggested local area unemployment [3, 7, 8] and socioeconomic gradient [17] as important contextual factors for overall DP, but compositional factors have mostly been identified as more significant for mental disorder sickness absence and DP rates than contextual factors [16, 18]. However, studies concerning regional differences in the Nordic countries have found associations between mental disorder DP rates and urban/rural environments [11, 12]. Access to mental health services and rehabilitation, the screening and recognition of mental disorders, local treatment practice, and the different effects of social fragmentation, migration, unemployment, and alcohol dependence on DP rates in different geographical settings have been suggested as possible explanations. A higher population density also appears to be connected with an overall increased risk of psychosis and mood disorders in the Swedish population [19]. However, one Norwegian study did not find associations between the provision of psychiatric care (represented by the number of staff and availability of beds) and regional differences in mental disorder DP [20].

Finland has greater sociodemographic and -economic differences between larger regions than between smaller residential neighborhoods [21, 22]. Distances are also long both between and within regions. There are well documented regional differences in health, and prior research in Finland has identified that geographical differences in sociodemographic and -economic structure are associated with differences in regional health, health behavior and mortality [22–26]. The more disadvantaged regions have poorer health outcomes, lower population density, longer distances between population centers as well as older average age and lower socioeconomic population distribution. These regions are centered in the eastern and northern parts of the country. The capital area, the southern regions and the western region of Ostrobothnia have traditionally better health outcomes, higher population density and an overall higher socioeconomic distribution [22, 25, 27–29]. Furthermore, the southern and western regions have a higher proportion of the Swedish-speaking minority, also associated with better health outcomes and lower DP as well as mortality rates. This health inequality has been explained by cultural differences as well as higher social capital in the Swedish-speaking minority [26, 28]. However, there is no significant regional variation reported in mental disorder prevalence in Finland, except in the case of psychotic disorders, which follow the previously mentioned differences [27, 30].

Finnish public health care services are divided into 21 hospital districts with a high degree of autonomy in providing the services in their area. Some districts arrange mental health outpatient visits in primary health care and some mainly in psychiatric special health care [31]. Rehabilitative psychotherapy has been available for most people within the scope of private services and is publicly reimbursed by the Social Insurance Institution of Finland. There is however substantial regional variation in the availability of rehabilitative psychotherapy services, as they are for the most part concentrated in the university hospital areas [31]. Previous studies have identified that the variation in diversity of mental health and substance abuse services is 84% explained by the area’s population size [32]. The hospital districts’ population size is not however directly related to the geographical size of the area. Notably, almost one third of the population of Finland is situated in the capital area, the hospital district of Helsinki and Uusimaa (HUS), whereas only about 1 % of the population lives in the smallest hospital district of East Savo. The hospital district of Lapland has the largest surface area of all the districts, but only a little over 2 % of the Finnish population reside in it.

In the Finnish DP system, the applicant is generally required to have had reduced working capacity and sickness benefits for 300 days before applying for DP. Medical insurance specialists then evaluate the application for temporary or permanent DP. The evaluation process is centralized nationally. Regarding mental disorder DP in Finland, research about its regional variance and district-level factors is scarce. One study found different regional mental disorder DP rates, with mood disorder DP having a greater regional variance than other mental disorder DP [28]. In the case of non-affective psychotic disorder DP, differences in regional DP rates have been reported which differ from regional differences in lifetime prevalence of psychotic disorders [27, 33]. These differences appear not to be explained by the region’s social indicators, for example the unemployment rate or health care spending. However, higher DP rates were associated with high involuntary treatment rates. This may reflect the influence of differences in regional treatment practices, which may be considered a source of regional inequality putting people living in different parts of Finland in unequal positions [33].

The current research literature lacks information on the contemporary regional mental disorder DP rate differences and the role of contextual and mental health service factors in this DP variation. The aim of this study was to investigate factors behind the regional differences in all mental disorder DP and separately in its two largest diagnostic groups in Finland, ICD-10 classifications F30–39 (mood disorders) DP and F20–29 (non-affective psychotic disorders) DP [34]. Our hypothesis was that regional differences would approximately follow the previously reported east-north – south-west divide in Finnish health outcomes [22, 25]. We also hypothesized that the district-level factors in the form of socioeconomic deprivation, employment, access to mental health services and local treatment practice would be associated with different regional DP risks. In this unique research setting we studied the regional effects of a wide range of contextual and mental health service factors on mental disorder DP using sophisticated statistical methods, while controlling for the compositional factors of the population. This study is part of the RETIRE – research project, which aims to study the risk factors and sequences of mental health-based disability pensioning and examine the effectiveness of service systems in different hospital districts in Finland [35, 36].

## Methods

### Data

The study subjects were all Finnish citizens granted a temporary, permanent, full or partial DP due to a mental disorder (ICD 10: F04-F69, F80-F99) for the first time between 2010 and 2015 (*N* = 50,728) [35]. The utilized data was gathered from the registers of the Social Insurance Institution of Finland, The Finnish Centre for Pensions, the Finnish Institute for Health and Welfare (THL) and Statistics Finland. The following subjects were omitted from the final data: 1) individuals with any previous or primarily somatic pensions; 2) individuals aged under 18 or over 65; 3) individuals who had moved to a new hospital district during the last three years before DP; 4) individuals living in the hospital district of the Åland Islands because of the district’s small sample size and divergent sample. The final data set included 36,879 subjects with mental disorder DP. For a more detailed analysis of our data please see our previous study [36]. We used the Finnish population as an exposure in our model: the information on the age, gender, occupational status and residential hospital district of the Finnish population aged 18 to 65 in 2015 was acquired from Statistics Finland (*N* = 2,991,434).

### Explanatory variables

This study’s compositional factors included age, gender, occupational status and region of residence by hospital district. Age was classified into five groups: 18–25 years, 26–35 years, 36–45 years, 46–55 years, and 56–65 years. Occupational status was classified into eight groups in line with Statistics Finland’s classification: blue-collar workers, lower white-collar workers, upper white-collar workers, entrepreneurs, agriculture and forestry entrepreneurs, students, unemployed and unknown occupational status. For a more detailed analysis of the demographics of DP the receivers please see our previous study [36].

District-level contextual and mental health service factors were collected from the year 2015 from the Sotkanet Indicator Bank, an information portal provided by THL that offers key population welfare and health data [37]. The six contextual sociodemographic and -economic factors studied were Swedish-speaking population as proportion of the total population; persons with foreign background per 1000 persons; general at-risk-of-poverty rate; employed as proportion of the total population; long-term unemployed as proportion of the labor force; and annual sale of alcoholic beverages per capita as liters of pure alcohol.

The nine mental health service factors used in the study were population density as population/km^2^ (used as a proxy indicator for the accessibility of mental health services); all mental health outpatient visits of adults per 1000 persons aged 18 and over (including both primary health care and psychiatric special health care visits); outpatient visits per 1000 persons in psychiatric units; mental health visits in primary health care per 1000 persons; recipients of rehabilitative psychotherapy per 1000 persons aged 18–64; involuntary referrals for observation in psychiatric inpatient care per 1000 persons aged 18–64; periods of care in psychiatric inpatient care per 1000 persons aged 18–64; patients in psychiatric inpatient care, per 1000 persons aged 18–64; and the number of care days in psychiatric inpatient care per 1000 persons aged 18–64.

The original continuous variables were transformed into categorical variables with four groups (named highest to lowest) using one standard deviation (SD) of each variable for the categorization. The lowest group included values smaller than one SD subtracted from the mean and the highest larger than one SD added to the mean (i.e. lowest: value < mean - SD; lower: mean - SD < value < mean; higher: mean < value < mean + SD; highest: value > mean + SD). Each hospital district was assigned to one of the groups in each district-level variable accordingly (Supplementary file 1). Three of the variables (Swedish-speaking population, persons with foreign background and population density) did not have values smaller than one SD subtracted from the mean, so no hospital districts were assigned to the lowest group in those variables.

### Statistical analysis

As the dependent variable is the number of events and the data was over-dispersed, we applied negative binomial regression analysis to study the levels of risk for mental disorder DP in the Finnish hospital districts. The negative binomial model was tested against other count models and found to be most suitable for this data. Incidence rate ratios (IRR) and 95% confidence intervals (95% CI) were calculated separately for the hospital districts for all mental disorder DP, mood disorder DP (*N* = 24,132) and non-affective psychotic disorder DP (*N* = 6171). The regression analyses were performed by applying robust standard errors, using the Finnish population as an exposure. First, the IRRs were analyzed using crude models to obtain the crude IRR for each hospital district (Supplementary file 2). Second, the compositional factors age, gender and occupational status were added to the analysis to obtain the adjusted IRRs for the hospital districts.

Furthermore, the district-level factors were added to the models in order to detect the associations between the contextual and mental health service factors and the rate of retiring. The models were compared with the Akaike information criterion (AIC) and Bayesian information criterion (BIC) scores and Pseudo R2 to determine the most suitable model. Due to the correlative nature of the district-level factors, they were added to the model one at a time. The correlation between the district-level factors is shown in Supplementary file 3. The models with the district-level factors were also adjusted on the basis of age, gender and occupational status. The crude models for district-level factor IRRs and 95% CI are described in Supplementary file 4. In additional analysis we also created a heatmap of the categorization of district-level factors, in which the district-level factors were clustered with Euclidean distance and Ward linkage. This demonstrates the complex nature and relationships of district-level factors and hospital district DP risk levels. Statistical analyses were conducted with Stata Version 16.0 and the illustration of the IRR in Finland was drawn with R and the packages gdalUtils, rgdal, tmap, and maptools [38–42].

## Results

### Regional variation in mental disorder DP

**Fig. 1:**
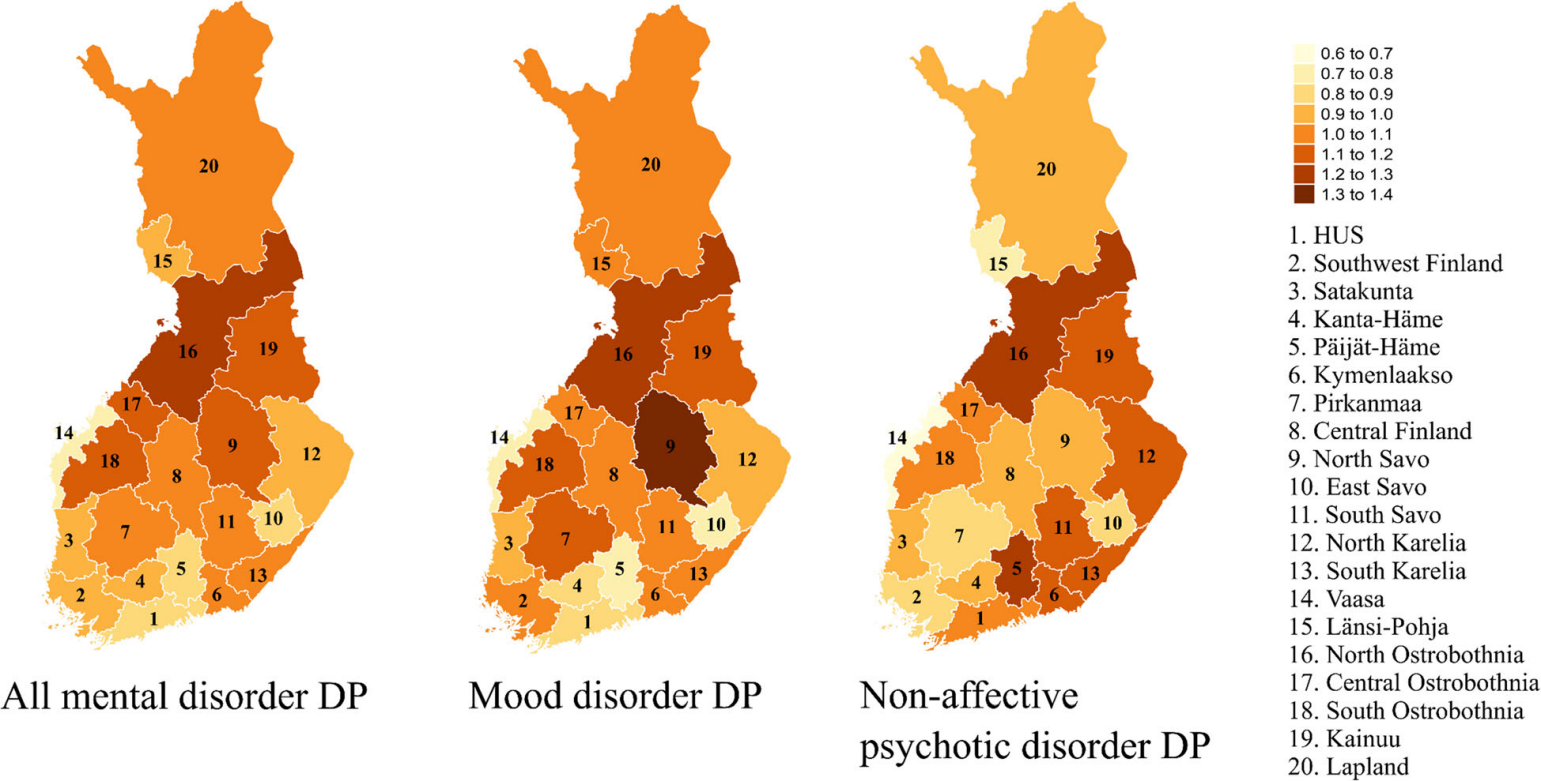
Hospital district differences in all mental disorder-related disability pensions (DP), mood disorder (F30–39) DP and non-affective psychotic disorder (F20–29) DP in Finland, 2010–2015 by incidence rate ratio (IRR). Negative binomial regression model adjusted based on the compositional factors gender, age and occupational status

Distinct differences between hospital districts in mental disorder DP and regional differences between mood disorder (F30–39) and non-affective psychotic disorder (F20–29) DP were observed. Hospital district differences adjusted for the compositional factors age, gender, and occupational status by IRR and 95% CI for all mental disorder DP, and separately for mood disorder and non-affective psychotic disorder DP, are described in Table 1 and Fig. 1.

**Table 1:** Hospital district differences between mental disorder–related disability pensions (DP)

	All mental disorder DP		Mood disorder DP		Non–affective psychotic disorder DP	
	IRR	95% CI	IRR	95% CI	IRR	95% CI
National mean	1.00		1.00		1.00	
Helsinki and Uusimaa (HUS)	0.85	0.78–0.93	0.84	0.78–0.90	1.09	0.93–1.27
Southwest Finland	0.96	0.89–1.04	1.03	0.95–1.11	0.87	0.77–0.99
Satakunta	0.97	0.89–1.06	0.99	0.90–1.09	0.93	0.80–1.08
Kanta–Häme	0.90	0.81–1.01	0.87	0.77–0.99	0.91	0.76–1.09
Päijät–Häme	0.86	0.78–0.95	0.78	0.70–0.86	1.29	1.10–1.50
Kymenlaakso	1.08	0.99–1.18	1.08	0.99–1.17	1.11	0.97–1.28
Pirkanmaa	1.02	0.94–1.11	1.11	1.03–1.20	0.87	0.75–1.00
Central Finland	1.01	0.91–1.11	1.06	0.97–1.16	0.99	0.86–1.15
North Savo	1.17	1.07–1.28	1.33	1.21–1.46	1.00	0.85–1.17
East Savo	0.86	0.72–1.03	0.70	0.59–0.84	0.88	0.65–1.18
South Savo	1.05	0.95–1.16	1.03	0.93–1.15	1.12	0.94–1.33
North Karelia	0.95	0.86–1.04	0.92	0.83–1.02	1.19	1.05–1.36
South Karelia	1.01	0.92–1.11	1.07	0.97–1.17	1.11	0.93–1.32
Vaasa	0.76	0.68–0.84	0.71	0.63–0.80	0.65	0.53–0.80
Länsi–Pohja	0.99	0.85–1.15	1.04	0.89–1.22	0.74	0.55–0.99
North Ostrobothnia	1.22	1.13–1.31	1.29	1.21–1.39	1.27	1.10–1.46
Central Ostrobothnia	1.13	0.95–1.35	1.04	0.93–1.16	1.02	0.81–1.29
South Ostrobothnia	1.15	1.05–1.25	1.19	1.07–1.32	1.09	0.92–1.28
Kainuu	1.19	1.05–1.36	1.18	1.02–1.36	1.16	0.93–1.44
Lapland	1.02	0.91–1.14	1.03	0.92–1.16	0.99	0.80–1.23
Nagelkerke Pseudo–R^2^	0.790		0.799		0.668	
AIC	9531.933		7930.831		5375.144	
BIC	9709.399		8108.298		5552.61	

Hospital district differences between all mental disorder–related disability pensions (DP), mood disorder (F30–39) DP and non–affective psychotic disorder (F20–29) DP in Finland, 2010–2015 by incidence rate ratio (IRR) and 95% confidence interval (95% CI).

Negative binomial regression model adjusted based on the compositional factors gender, age and occupational status

A higher risk of overall mental disorder DP compared to the national level of risk was found in the hospital districts of North Savo (IRR 1.17; 95% CI 1.07–1.28), North Ostrobothnia (IRR 1.22; 95% CI 1.13–1.31), South Ostrobothnia (IRR 1.15; 95% CI 1.05–1.25) and Kainuu (IRR 1.19; 95% CI 1.05–1.36). A lower risk of mental disorder DP was found in HUS (IRR 0.85; 95% CI 0.78–0.93), Päijät-Häme (IRR 0.86; 95% CI 0.78–0.95) and Vaasa (IRR 0.76; 95% CI 0.68–0.84). Regarding diagnostic categories, a higher risk for mood disorder DP was found in Pirkanmaa (IRR 1.11; 95% CI 1.03–1.20), North Savo (IRR 1.33; 95% CI 1.21–1.46), North Ostrobothnia (IRR 1.29; 95% CI 1.21–1.39), South Ostrobothnia (IRR 1.19; 95% CI 1.07–1.32) and Kainuu (IRR 1.18; 95% CI 1.02–1.36), and a lower risk in HUS (IRR 0.84; 95% CI 0.78–0.90), Päijät-Häme (IRR 0.78; 95% CI 0.70–0.86), East Savo (IRR 0.70; 95% CI 0.59–0.84) and Vaasa (IRR 0.71; 95% CI 0.63–0.80), as well as a slight indication of lower risk in Kanta-Häme (IRR 0.87; 95% CI 0.77–0.99). In the case of non-affective psychotic disorder DP, a higher risk was found in Päijät-Häme (IRR 1.29; 95% CI 1.10–1.50), North Karelia (IRR 1.19; 95% CI 1.05–1.36) and North Ostrobothnia (IRR 1.27; 95% CI 1.10–1.46). By contrast, a lower risk was found in Vaasa (IRR 0.65; 95% CI 0.53–0.80) and some indications of a lower risk were recorded in Southwest Finland (IRR 0.87; 95% CI 0.77–0.99) and Länsi-Pohja (IRR 0.74; 95% CI 0.55–0.99). Only the hospital district of North Ostrobothnia had a higher risk and the hospital district of Vaasa had a lower risk of DP in all three diagnostic categories compared to the national mean. Notably, the hospital district of Päijät-Häme had a distinct pattern of DP with a higher risk of non-affective psychotic disorder DP (IRR 1.29; 95% CI 1.10–1.50) but a lower risk of mood disorder DP (IRR 0.78; 95% CI 0.70–0.86) compared to the Finnish national mean of DP risk. Furthermore, the hospital districts of HUS, Pirkanmaa, North Savo and North Karelia differed in their risks of mood disorder DP and non-affective psychotic disorder DP: HUS and North Karelia had a higher risk of non-affective psychotic disorder DP than mood disorder DP, whereas Pirkanmaa and North Savo had a higher risk of mood disorder DP than non-affective psychotic disorder DP.

### District-level factors

**Fig. 2:**
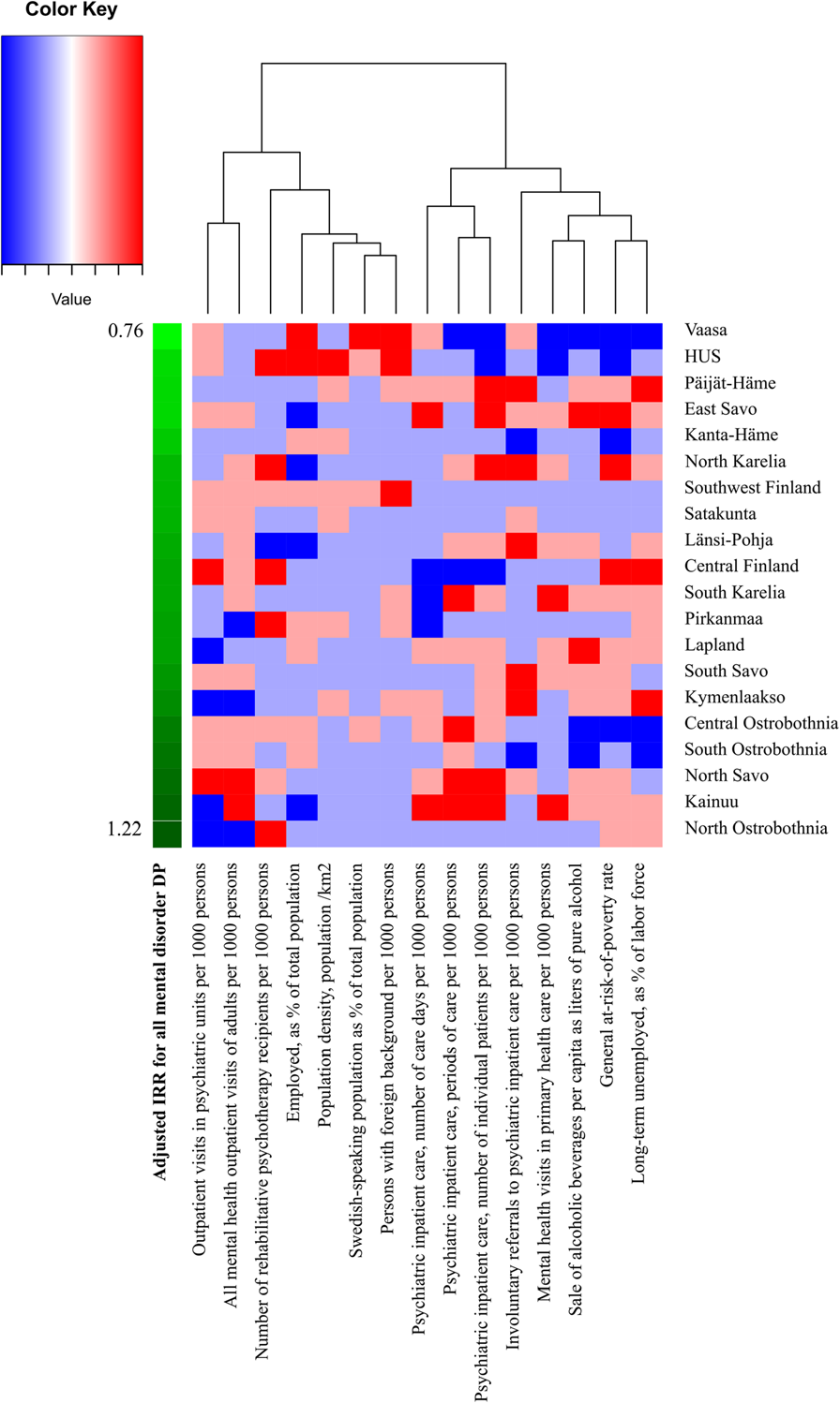
Categorization of hospital districts to all district-level factors. Heatmap rows are sorted based on the IRR of the districts, and columns are clustered with Euclidean distance and Ward linkage. (4 = highest, 3 = higher, 2 = lower, 1 = lowest)

The categorization of hospital districts to the district-level factors and the association of district-level factors with hospital districts and DP risk levels can be seen in Fig. 2. The heatmap rows are sorted based on the IRR of the districts. The IRR and 95% CI calculated for district-level contextual factors (adjusted for the compositional factors age, gender and occupational status) are shown in Table 2. The highest rates of Swedish-speaking and foreign background population were associated with a lower level of regional risk for all mental disorder (Swedish-speaking population IRR 0.84; 95% CI 0.78–0.90 and foreign background population IRR 0.90; 95% CI 0.86–0.93) and mood disorder DP (Swedish-speaking population IRR 0.79; 95% CI 0.73–0.86 and foreign background population IRR 0.89; 95% CI 0.85–0.92) compared to the national mean. The Swedish-speaking population’s highest rate was also associated with a lower non-affective psychotic disorder DP risk (IRR 0.74; 95% CI 0.63–0.85). Vaasa was however the only hospital district included in the highest rate of the Swedish-speaking population.

**Table 2:** Associations of regional differences in contextual factors for mental disorder-related disability pensions (DP)

	All mental disorder DP		Mood disorder DP		Non-affective psychotic disorder DP	
	IRR	95% CI	IRR	95% CI	IRR	95% CI
Swedish-speaking population as % proportion of total population	*p* < 0.001		*p* < 0.001		*p* < 0.001	
Highest	0.84	0.78–0.90	0.79	0.73–0.86	0.74	0.63–0.85
Higher	1.04	0.99–1.11	1.05	1.00–1.11	1.14	1.03–1.26
Lower	1.14	1.09–1.20	1.20	1.14–1.26	1.19	1.09–1.29
Lowest	–NA		–NA		–NA	
Persons with foreign background per 1000 persons	*p* < 0.001		*p* < 0.001		*p* = 0.115	
Highest	0.90	0.86–0.93	0.89	0.85–0.92	0.93	0.86–1.00
Higher	1.03	0.99–1.07	1.03	0.99–1.07	1.05	0.98–1.12
Lower	1.09	1.05–1.12	1.10	1.06–1.13	1.03	0.98–1.09
Lowest	–NA		–NA		–NA	
General at-risk-of-poverty rate	*p* < 0.001		*p* < 0.001		*p* < 0.001	
Highest	0.98	0.92–1.03	0.96	0.91–1.02	1.04	0.96–1.13
Higher	1.10	1.06–1.14	1.12	1.08–1.17	1.13	1.06–1.20
Lower	1.04	1.00–1.08	1.09	1.04–1.13	0.89	0.84–0.95
Lowest	0.90	0.85–0.95	0.85	0.81–0.89	0.95	0.88–1.03
Employed, as proportion% of total population	*p* < 0.001		*p* < 0.001		*p* = 0.003	
Highest	0.84	0.80–0.90	0.82	0.78–0.87	0.96	0.87–1.06
Higher	1.05	1.01–1.10	1.08	1.04–1.13	0.92	0.86–0.99
Lower	1.09	1.05–1.13	1.13	1.08–1.17	1.09	1.03–1.16
Lowest	1.03	0.98–1.09	1.00	0.94–1.06	1.03	0.95–1.13
Long-term unemployed, as proportion% of labor force	*p* = 0.056		*p* = 0.003		*p* = 0.017	
Highest	0.98	0.93–1.03	0.96	0.92–1.01	1.11	1.03–1.20
Higher	1.05	1.01–1.09	1.08	1.04–1.12	1.04	0.97–1.11
Lower	0.97	0.94–1.01	1.00	0.96–1.04	0.98	0.91–1.04
Lowest	1.00	0.94–1.06	0.97	0.91–1.03	0.89	0.81–0.98
Sale of alcoholic beverages per capita, as liters of pure alcohol	*p* = 0.102		*p* = 0.014		*p* = 0.038	
Highest	0.96	0.89–1.04	0.92	0.85–1.00	0.97	0.84–1.11
Higher	1.05	1.00–1.10	1.08	1.03–1.13	1.10	1.02–1.19
Lower	0.99	0.95–1.03	1.02	0.98–1.07	1.03	0.96–1.10
Lowest	1.00	0.94–1.07	0.98	0.92–1.04	0.91	0.82–1.01

Associations of regional differences in contextual factors for all mental disorder-related disability pensions (DP), mood disorder (F30–39) DP and non-affective psychotic disorder (F20–29) DP in Finland by incidence rate ratio (IRR) and 95% confidence interval (95% CI) (national mean as reference: 1.00).

Negative binomial regression model adjusted based on the compositional factors gender, age and occupational status

The lowest general at-risk-of-poverty rates were associated with a lower regional risk of all mental (IRR 0.90; 95% CI 0.85–0.95) and mood (IRR 0.85; 95% CI 0.81–0.89) disorder DP compared to the national mean. In the case of non-affective psychotic disorder DP, a lower general at-risk-of-poverty rate indicated a lower risk (IRR 0.89; 95% CI 0.84–0.95). In all three diagnostic categories the higher, but interestingly not the highest, general at-risk-of-poverty rate indicated a higher risk of DP compared to the national mean. The hospital districts HUS, Kanta-Häme, Vaasa and Central Ostrobothnia had the lowest at-risk-of-poverty rates.

With the highest employment rate, the hospital districts HUS and Vaasa had lower regional risk levels in all mental (IRR 0.84; 95% CI 0.80–0.90) and mood (IRR 0.82; 95% CI 0.78–0.87) disorder DP compared to the national mean. The highest rates of long-term unemployment were associated with a higher regional risk of non-affective psychotic disorder DP compared to the national mean(IRR 1.11; 95% CI 1.03–1.20). Furthermore, the lowest rates of long-term unemployment were associated with a lower regional non-affective psychotic disorder DP risk (IRR 0.89; 95% CI 0.81–0.98). Concerning the regional alcoholic beverage sale rate there was a higher risk of mood (IRR 1.08; 95% CI 1.03–1.13) and non-affective psychotic disorder DP (IRR 1.10; 95% CI 1.02–1.19) associated with the higher, but not the highest, regional sale rate compared to the national mean.

The IRR and 95% CI calculated for mental health service factors (adjusted for the compositional factors age, gender and occupational status) are shown in Table 3. In the case of population density, (a proxy for the accessibility of mental health services), there was a lower risk associated with the highest population density rate in all mental disorder DP (IRR 0.89; 95% CI 0.84–0.96) and mood disorder DP (IRR 0.87; 95% CI 0.83–0.92), as well as a higher risk of DP associated with a lower population density in all mental disorder DP (IRR 1.10; 95% CI 1.06–1.14) and mood disorder DP (IRR 1.12; 95% CI 1.08–1.16) compared to the national mean. It is noteworthy that HUS was the only hospital district that was accounted as having the highest ratio of population density, with half of the other districts categorized as having the lower population density rate. No hospital district was categorized as having the lowest rate of population density, as none had values smaller than one SD subtracted from the mean.

**Table 3:** Associations of regional differences in mental health service factors for mental disorder-related disability pensions (DP)

	All mental disorder DP		Mood disorder DP		Non-affective psychotic disorder DP	
	IRR	95% CI	IRR	95% CI	IRR	95% CI
Population density, population/km^2^ (proxy for the accessibility of treatment)	*p* < 0.001		*p* < 0.001		*p* = 0.200	
Highest	0.89	0.84–0.96	0.87	0.83–0.92	1.06	0.95–1.18
Higher	1.02	0.98–1.06	1.03	0.99–1.07	0.94	0.88–1.01
Lower	1.10	1.06–1.14	1.12	1.08–1.16	1.00	0.94–1.07
Lowest	NA–		NA–		NA–	
All mental health outpatient visits of adults per 1000 persons	p < 0.001		p < 0.001		*p* = 0.628	
Highest	1.14	1.07–1.21	1.20	1.13–1.28	1.01	0.91–1.12
Higher	0.97	0.94–1.01	0.96	0.93–1.00	0.97	0.91–1.03
Lower	0.84	0.81–0.88	0.79	0.75–0.82	0.99	0.92–1.07
Lowest	1.07	1.02–1.12	1.10	1.05–1.14	1.04	0.96–1.12
Outpatient visits in psychiatric units per 1000 persons	*p* < 0.001		*p* < 0.001		*p* = 0.014	
Highest	1.05	1.00–1.11	1.13	1.07–1.19	0.96	0.88–1.05
Higher	0.93	0.89–0.97	0.89	0.86–0.92	0.94	0.88–1.00
Lower	0.93	0.89–0.97	0.91	0.88–0.95	0.99	0.93–1.06
Lowest	1.10	1.05–1.15	1.09	1.05–1.14	1.12	1.04–1.21
Mental health visits in primary health care per 1000 persons	*p* < 0.001		*p* < 0.001		*p* = 0.471	
Highest	1.12	1.05–1.19	1.13	1.06–1.21	1.09	0.97–1.22
Higher	1.00	0.95–1.06	0.98	0.93–1.04	0.99	0.91–1.08
Lower	1.07	1.03–1.11	1.10	1.06–1.14	0.99	0.93–1.05
Lowest	0.84	0.79–0.89	0.81	0.77–0.86	0.94	0.84–1.04
Number of rehabilitative psychotherapy recipients per 1000 persons	*p* = 0.125		*p* < 0.001		*p* = 0.027	
Highest	0.99	0.94–1.05	0.99	0.94–1.05	1.15	1.04–1.27
Higher	1.06	1.00–1.13	1.09	1.03–1.16	1.01	0.90–1.12
Lower	0.97	0.92–1.02	0.93	0.88–0.97	1.09	1.00–1.20
Lowest	0.98	0.87–1.10	1.00	0.88–1.13	0.79	0.62–0.99
Involuntary referrals to psychiatric inpatient care per 1000 persons	*p* = 0.145		*p* = 0.010		*p* < 0.001	
Highest	0.98	0.94–1.02	0.95	0.90–0.99	1.13	1.05–1.21
Higher	0.96	0.92–1.01	0.98	0.93–1.04	0.87	0.80–0.94
Lower	1.03	0.99–1.07	1.05	1.01–1.09	1.03	0.96–1.09
Lowest	1.03	0.97–1.09	1.02	0.96–1.10	1.00	0.90–1.10
Psychiatric inpatient care, periods of care per 1000 persons	*p* < 0.001		*p* < 0.001		*p* = 0.004	
Highest	1.13	1.07–1.19	1.16	1.11–1.22	1.06	0.97–1.15
Higher	0.99	0.95–1.04	0.97	0.92–1.02	1.11	1.03–1.19
Lower	1.00	0.96–1.04	1.00	0.96–1.04	1.01	0.95–1.08
Lowest	0.89	0.84–0.95	0.89	0.83–0.95	0.84	0.76–0.93
Psychiatric inpatient care, number of individual patients per 1000 persons	*p* < 0.001		*p* = 0.004		*p* = 0.048	
Highest	1.02	0.98–1.07	1.01	0.96–1.06	1.10	1.02–1.18
Higher	1.06	1.01–1.10	1.05	1.01–1.09	1.01	0.94–1.08
Lower	1.05	1.01–1.09	1.09	1.05–1.13	0.96	0.90–1.02
Lowest	0.88	0.84–0.93	0.87	0.83–0.91	0.95	0.87–1.03
Psychiatric inpatient care, number of care days per 1000 persons	*p* = 0.806		*p* = 0.082		*p* = 0.484	
Highest	1.04	0.95–1.13	0.96	0.87–1.06	1.03	0.89–1.19
Higher	0.98	0.94–1.03	0.98	0.93–1.03	1.00	0.92–1.08
Lower	0.98	0.94–1.03	1.00	0.96–1.04	1.03	0.96–1.10
Lowest	1.00	0.95–1.05	1.06	1.01–1.12	0.94	0.87–1.03

Associations of regional differences in mental health service factors for all mental disorder-related disability pensions (DP), mood disorder (F30–39) DP and non-affective psychotic disorder (F20–29) DP in Finland by incidence rate ratio (IRR) and 95% confidence interval (95% CI) (national mean as reference: 1.00)

Negative binomial regression model adjusted based on the compositional factors gender, age and occupational status

In all adult mental health outpatient visits (including both visits in psychiatric units and in primary health care), the highest and lowest numbers of visits were associated with a higher regional risk of DP in all mental (highest: IRR 1.14; 95% CI 1.07–1.21; lowest: IRR 1.07 95% CI 1.02–1.12) and mood disorders (highest: IRR 1.20; 95% CI 1.13–1.28; lowest: IRR 1.10 95% CI 1.05–1.14). North Savo and Kainuu had the highest numbers of visits, and Kymenlaakso, Pirkanmaa and North Ostrobothnia had the lowest. The same association of higher regional risk with the lowest number of visits was also discernable in outpatient visits in psychiatric units in all three diagnostic categories, but the highest number of visits was associated only with a higher risk of mood disorder DP (IRR 1.13; 95% CI 1.07–1.19) compared to the national mean. In primary health care mental health visits, the lowest rate of visits, in HUS and Vaasa, showed a lower risk of all mental (IRR 0.84; 95% CI 0.79–0.89) and mood disorder DP (IRR 0.81; 95% CI 0.77–0.86), while the highest rate of visits, in South Karelia and Kainuu, was associated with a higher risk. There was no association between the regional risk of all mental disorder DP and the number of recipients of rehabilitative psychotherapy. However, in the case of mood disorder DP the higher number of recipients was associated with a higher regional risk (IRR 1.09; 95% CI 1.03–1.16) and the lower number with a lower risk (IRR 0.93; 95% CI 0.88–0.97), and in non-affective psychotic disorder DP the highest number of recipients was associated with a higher risk (IRR 1.15; 95% CI 1.04–1.27) and the lowest number showed some indication of lower risk (IRR 0.79; 95% CI 0.62–0.99).

In terms of psychiatric inpatient care, involuntary referrals for inpatient care showed no association with regional all mental disorder DP risk. In mood disorder DP, the highest numbers of involuntary referrals had a slight indication of a lower DP risk (IRR 0.95; 95% CI 0.90–0.99) compared to the national mean, and in non-affective psychotic disorder DP the highest number of referrals was associated with a higher DP risk (IRR 1.13; 95% CI 1.05–1.21) compared to the national mean. In the case of hospitalization periods, a lower regional risk of DP for all three diagnostic categories was associated with the lowest number of care periods and a higher risk was associated with the highest number of care periods in all mental (IRR 1.13; 95% CI 1.07–1.19) and mood disorders (IRR 1.16; 95% CI 1.11–1.22) compared to the national mean. The lowest numbers of inpatient care patients were also associated with a lower regional risk of DP in all mental (IRR 0.88; 95% CI 0.84–0.93) and mood disorder DP (IRR 0.87; 95% CI 0.83–0.91) compared to the national mean, and the highest numbers of individual patients with a higher risk of DP for non-affective psychotic disorders (IRR 1.10; 95% CI 1.02–1.18). There was no association between the regional risk of DP and the number of days in psychiatric inpatient care.

## Discussion

In this comprehensive population-level study, we found significant differences between Finnish hospital districts in disability pensioning for mental disorders even after adjusting for the compositional background differences in the populations of the different districts. The hospital districts with the lowest all mental disorder DP risk were HUS, which includes and surrounds the capital area; Vaasa in the west and Päijät-Häme in the south. A higher regional risk of DP was found in the North Savo and Kainuu hospital districts in the east and in the Ostrobothnia districts in the north-western regions (excluding the Vaasa and Central Ostrobothnia hospital districts). We identified several district-level factors that were associated with these differences, and some that surprisingly were not. Overall, the regional differences in mental disorder DP do not appear to completely follow the traditional north-east – south-west -division of health differences described in the earlier research literature [22, 25, 27, 29]. Furthermore, the regional differences were not explained purely by sociodemographic and -economic differences. This points towards the role of structural or functional differences in service systems and rehabilitative processes.

Compared to the mental disorder DP risks described in two prior Finnish studies [28, 33], our results were similar but with some regional differences, which were probably due to the different time frame of these studies. As a novel finding, we reported several hospital districts with differences in mood disorder DP compared to the national mean and hospital districts, with an opposite pattern of mood/non-affective psychotic disorder DP in their district. Because the regional differences in mental disorder DP did not disappear after adjusting for compositional effects, the reason for these differences can be suspected to be elsewhere, most probably in the service system. One possibility proposed by Kiviniemi et al. (2011) is that the regional DP variance reflects differences in mental health service systems and local treatment practices. The effectiveness of the service system in responding to the population’s mental health service needs with effectual and timely treatment can be seen as a crucial element in preventing work disability and DPs, especially in the case of mood disorder DP.

### Contextual factors

We found several district-level contextual factor groups associated with regional mental disorder DP risk differences. Indicators of higher regional socioeconomic level, such as particularly low poverty rates and high employment rates, were associated with lower regional all mental and mood disorder DP risk. The hospital districts of the capital area HUS and western Vaasa were prominent in these factor groups, which could partly explain their lower risk of mental disorder DP. HUS and Vaasa also had the highest rates of Swedish-speaking and foreign background population, which was also associated with a lower DP risk. There is prior literature attesting to the health advantages of the Swedish speaking minority in Finland. Explanations suggested earlier have been cultural differences and a high quantity of social capital, but the reasons are complex and our results in this study most probably reflect on the interrelations between different contextual factors [26, 28].

The sale of alcoholic beverages per capita appeared to have no clear association with mental disorder DP, which was unexpected. However, it is worthy of note that the regional rate of alcohol sales is not equivalent to alcohol consumption rates, because some alcohol may have been sold in a different hospital district area or brought from abroad. Thus, the possible associations between accurate alcohol consumption, the possible prevalence of alcohol dependence and mental disorder DP are not necessarily registered by the regional sales figures of alcoholic beverage rates per capita included in this study.

### Mental health service factors

Firstly, highest and lower population density showed an association with regional overall mental and mood disorder DP risk, but not with non-affective psychotic disorder DP risk. This may be related to easier accessibility of mental health services and better treatment outcomes regarding working ability with mood disorders. There is also evidence of more diverse mental health and substance abuse services with increasing population size of the area [32].

Concerning outpatient services, we identified an indication of a connection between all mental health adult outpatient visits (including both visits in psychiatric units and in primary health care) and regional all mental disorder and mood disorder DP risk: both the regionally highest and lowest numbers of outpatient visits showed an association with a higher risk of DP compared to numbers of outpatient visits closer to the national average. Possible explanations for the highest rate of visits’ higher risk could include inefficient district service systems or worse mental health circumstances, but because of the complexity of the multicausal relations associated with regional DP risk (as seen in Fig. 2), it is not possible to deduce this in the scope of this study. However a previous study has shown that there are no clear regional differences between mood disorder prevalence in Finland [30]. On the other end of the spectrum, possible explanations for the high regional risk of DP for the lowest number of outpatient visits could include a regional relative lack of adequate outpatient services or more rural districts with less working opportunities, therefore making it a more frequent practice to grant DP in these districts rather than rehabilitation related to work. Interestingly the three hospital districts with the lowest visits (Kymenlaakso, Pirkanmaa and North Ostrobothnia) are not particularly rural nor do they have a particularly low employment rates.

We also studied psychiatric special health care and primary health care visits separately. A major difference in primary health care compared to special health care was that the regional lowest number of visits was however associated with a lower regional risk of mental disorder and mood disorder DP compared to the national mean. HUS and Vaasa were the two low-risk hospital districts with the lowest numbers of primary health care visits. The reasons for the low DP risk of these hospital districts most probably include complex interactions between the contextual and mental health service factors and cannot be unambiguously explained with the low number of primary health care visits. Nonetheless, different hospital districts have organized their mental health outpatient services differently between psychiatric special care and primary health care [31]. The service systems of HUS and Vaasa, with a higher rate of psychiatric special care-level services, might be more efficient in preventing mental disorder DP in their respective population.

The number of recipients of rehabilitative psychotherapy appeared to have no clear association with mental disorder DP in this study. This raises concerns about the efficiency of the provision of rehabilitative psychotherapy in Finland. Because its primary purpose is to improve and uphold rehabilitation clients’ ability to work and study, one would expect it to be associated with regional DP risk [31]. A possible reason may be that rehabilitative psychotherapy in Finland is not successfully used as preventive rehabilitation: it could begin too late to prevent a first time DP. It could also be focused on the people or districts not in the highest DP risk, as there is substantial regional variation in the availability of rehabilitative psychotherapy services.

In terms of psychiatric inpatient care, prior research has identified that the increased risk for non-affective psychotic disorder DP is associated with high involuntary treatment rates [33]. Our study gave an indication towards this same finding with the highest rate of involuntary referrals to psychiatric inpatient care. The lowest regional number of treatment periods was associated with a lower regional risk of mental disorder DP in all three diagnostic categories compared to the national mean. In addition, the lowest number of individual patients had an association with a lower regional DP risk in all mental and mood disorders, and in non-affective psychotic disorder DP the highest number of patients with a higher risk. This could indicate on the one hand that districts with an incidence of more severe disorders requiring inpatient care also naturally have a higher regional DP risk, or on the other hand that more inpatient- and hospital treatment-centered service systems are associated with a higher risk of DP compared to hospital districts with a stronger focus on outpatient services. However, we found no association with the number of care days in inpatient care. A higher number of care days may be expected to be associated with a higher rate of more severe mental disorders or less efficient mental health services and thus DP, but this appeared not to be the case in this study.

### Strengths, limitations and future research

The strengths of this study include the use of comprehensive, high coverage national-level data registers together with whole-population data. Finnish registers are of high quality, allowing the detailed and extensive research of different compositional and district-level factors and their associations with DP in this study [43, 44]. To our knowledge, there has not previously been any such extensive study of mental disorder DP concerning this broad range of different compositional, contextual, and mental health service factors in regional research of mental disorder DP.

One major limitation in this study was that due to the correlative nature of the district-level factors, they could only be added to the statistical models one at a time. Furthermore, we could not adjust the hospital district DP risks with the contextual and mental health service factors in addition to the compositional factors in order to study their effects on individual hospital district risks. In addition, because of the small number of hospital districts in the analyses, it is possible that some higher or lower risk levels in district-level factor groups could be attributed to specific hospital districts causing the different levels of risk as a data artefact. This would mean that in those assumed cases the differences in the contextual and mental health service factors in question would not be directly responsible for the different risk levels, but the hospital district would be acting as a mediator in the model and the true factors affecting the risk level would be elsewhere. In future research it would be important to study in greater depth the effects that mental health service systems and treatment practices have on regional mental disorder DP outcomes.

## Conclusions

In this comprehensive population-level study, we found evidence of significant regional variation and disparity in mental disorder DP and related contextual and mental health service factors, even when controlled for compositional factors. This variation appears not to be fully convergent with the traditional health differences in Finnish regions and may partly relate to differences in mental health service systems and local treatment practices. This may be considered as a source of regional inequality. A focus on the regional differences and the supply of mental health services appears to be indicated as one way to decrease mental disorder DP: regional mental health service systems and local treatment practices should adapt to the needs of the local population for optimal performance.

## Supplementary Information

**Additional file 1: Appendix 1.** The categorization of hospital districts to district-level contextual and mental health service factors using one standard deviation (SD) of each variable for the categorization.

**Additional file 2: Appendix 2.** Hospital district differences between all mental disorder–related disability pensions (DP), mood disorder (F30–39) DP and non–affective psychotic disorder (F20–29) DP in Finland, 2010–2015 by incidence rate ratio (IRR) and 95% confidence interval (95% CI). Crude model: Negative binomial regression model for hospital districts only.

**Additional file 3: Appendix 3.** The correlation between the district-level factors.

**Additional file 4: Appendix 4.** Associations of regional differences in contextual and mental health service factor for all mental disorder-related disability pensions (DP), mood disorder (F30–39) DP and non-affective psychotic disorder (F20–29) DP in Finland by incidence rate ratio (IRR) and 95% confidence interval (95% CI) (national mean as reference: 1.00)

## Data Availability

The used DP recipient and population datasets are available from the Social Insurance Institution of Finland, The Finnish Centre for Pensions, THL and Statistics Finland but restrictions apply to the availability of these data, which were used under license for the current study, and so are not publicly available. The district-level factors are publicly available from the Sotkanet Indicator Bank, an information portal provided by THL, and the datasets used and analyzed during the current study are available from the corresponding author on reasonable request.

## Abbreviations

AIC: Akaike information criterion
BIC: Bayesian information criterion
CI: Confidence Interval
DP: Disability pension
GDP: Gross domestic product
HUS: Hospital district of Helsinki and Uusimaa
IRR: Incidence rate ratio
SD: Standard deviation
THL: Finnish Institute for Health and Welfare
