# Supplementary material for: Contextual and mental health service factors in mental disorder-based disability pensioning in Finland – a regional comparison
Source: BMC Health Serv Res. 2021 Oct 11;21:1081. doi: 10.1186/s12913-021-07099-4 (PMC8507374; doi:10.1186/s12913-021-07099-4)
Supplement: Supplementary file 1 — Additional file 1: Appendix 1. The categorization of hospital districts to district-level contextual and mental health service factors using one standard deviation (SD) of each variable for the categorization. [file 12913_2021_7099_MOESM1_ESM.docx]

**Appendix 1.** The categorization of hospital districts to district-level contextual and mental health service factors using one standard deviation (SD) of each variable for the categorization.

Lowest: value < mean - SD; lower: mean - SD < value < mean; higher: mean < value < mean + SD; highest: value > mean + SD.

| District-level factors | Highest | Higher | Lower | Lowest |
| --- | --- | --- | --- | --- |
| Swedish-speaking population as % of total population | Vaasa | HUS  Southwest Finland  Central Ostrobothnia | Satakunta  Kanta-Häme  Päijät-Häme  Kymenlaakso  Pirkanmaa  Central Finland  North Savo  East Savo  South Savo  North Karelia  South Karelia  Länsi-Pohja  North Ostrobothnia  South Ostrobothnia  Kainuu  Lapland | None |
| Persons with foreign background per 1000 persons | HUS  Southwest Finland  Vaasa | Päijät-Häme  Kymenlaakso  Pirkanmaa  South Karelia | Satakunta  Kanta-Häme  Central Finland  North Savo  East Savo  South Savo  North Karelia  Länsi-Pohja  North Ostrobothnia  Central Ostrobothnia  South Ostrobothnia  Kainuu  Lapland | None |
| General at-risk-of-poverty rate | Central Finland  East Savo  North Karelia | Päijät-Häme  Kymenlaakso  North Savo  South Savo  South Karelia  North Ostrobothnia  Kainuu  Lapland | Southwest Finland  Satakunta  Pirkanmaa  Länsi-Pohja  South Ostrobothnia | HUS  Kanta-Häme  Vaasa  Central Ostrobothnia |
| Employed, as % of total population | HUS  Vaasa | Southwest Finland  Kanta-Häme  Pirkanmaa  Central Ostrobothnia  South Ostrobothnia  Lapland | Satakunta  Päijät-Häme  Kymenlaakso  Central Finland  North Savo  South Savo  South Karelia  North Ostrobothnia | East Savo  North Karelia  Länsi-Pohja  Kainuu |
| Long-term unemployed, as % of labor force | Päijät-Häme  Kymenlaakso  Central Finland | Pirkanmaa  East Savo  North Karelia  South Karelia  Länsi-Pohja  North Ostrobothnia  Kainuu  Lapland | HUS  Southwest Finland  Satakunta  Kanta-Häme  North Savo  South Savo | Vaasa  Central Ostrobothnia  South Ostrobothnia |
| Sale of alcoholic beverages per capita, as liters of pure alcohol | East Savo  Lapland | Päijät-Häme  Kymenlaakso  North Savo  South Savo  South Karelia  Länsi-Pohja  Kainuu | HUS  Southwest Finland  Satakunta  Kanta-Häme  Pirkanmaa  Central Finland  North Karelia  North Ostrobothnia | Vaasa  Central Ostrobothnia  South Ostrobothnia |
| Population density, population/km²  (proxy for the accessibility of treatment) | HUS | Southwest Finland  Satakunta  Kanta-Häme  Päijät-Häme  Kymenlaakso  Pirkanmaa | Central Finland  North Savo  East Savo  South Savo  North Karelia  South Karelia  Vaasa  Länsi-Pohja  North Ostrobothnia  Central Ostrobothnia  South Ostrobothnia  Kainuu  Lapland | None |
| All mental health outpatient visits of adults per 1000 persons | North Savo  Kainuu | Southwest Finland  Satakunta  Central Finland  East Savo  South Savo  North Karelia  South Karelia  Länsi-Pohja  Central Ostrobothnia  South Ostrobothnia | HUS  Kanta-Häme  Päijät-Häme  Vaasa  Lapland | Kymenlaakso  Pirkanmaa  North Ostrobothnia |
| Outpatient visits in psychiatric units per 1000 persons | Central Finland  North Savo | HUS  Southwest Finland  Satakunta  East Savo  South Savo  Vaasa  Central Ostrobothnia  South Ostrobothnia | Kanta-Häme  Päijät-Häme  Pirkanmaa  North Karelia  South Karelia  Länsi-Pohja | Kymenlaakso  North Ostrobothnia  Kainuu  Lapland |
| Mental health visits in primary health care per 1000 persons | South Karelia  Kainuu | East Savo  South Savo  North Karelia  Länsi-Pohja  Lapland | Southwest Finland  Satakunta  Kanta-Häme  Päijät-Häme  Kymenlaakso  Pirkanmaa  Central Finland  North Savo  North Ostrobothnia  Central Ostrobothnia  South Ostrobothnia | HUS  Vaasa |
| Rehabilitative psychotherapy, recipients per 1000 persons | HUS  Pirkanmaa  Central Finland  North Karelia  North Ostrobothnia | Southwest Finland  North Savo  Central Ostrobothnia | Satakunta  Kanta-Häme  Päijät-Häme  Kymenlaakso  East Savo  South Savo  South Karelia  Vaasa  South Ostrobothnia  Kainuu  Lapland | Länsi-Pohja |
| Involuntary referrals to psychiatric inpatient care per 1000 persons | Päijät-Häme  Kymenlaakso  South Savo  North Karelia  Länsi-Pohja | Satakunta  North Savo  East Savo  Vaasa | HUS  Southwest Finland  Pirkanmaa  Central Finland  South Karelia  North Ostrobothnia  Central Ostrobothnia  Kainuu  Lapland | Kanta-Häme  South Ostrobothnia |
| Psychiatric inpatient care, periods of care per 1000 persons | North Savo  South Karelia  Central Ostrobothnia  Kainuu | Päijät-Häme  North Karelia  South Ostrobothnia  Länsi-Pohja  Lapland | HUS  Southwest Finland  Satakunta  Kanta-Häme  Kymenlaakso  Pirkanmaa  East Savo  South Savo  North Ostrobothnia | Central Finland  Vaasa |
| Psychiatric inpatient care, number of individual patients per 1000 persons | Päijät-Häme  North Savo  East Savo  North Karelia  Kainuu | Kymenlaakso  South Savo  South Karelia  Central Ostrobothnia  Länsi-Pohja  Lapland | Southwest Finland  Satakunta  Kanta-Häme  Pirkanmaa  North Ostrobothnia  South Ostrobothnia | HUS  Central Finland  Vaasa |
| Psychiatric inpatient care, number of care days per 1000 persons | East Savo  Kainuu | Päijät-Häme  Kymenlaakso  North Savo  Vaasa  Central Ostrobothnia  Lapland | HUS  Southwest Finland  Satakunta  Kanta-Häme  South Savo  North Karelia  Länsi-Pohja  North Ostrobothnia  South Ostrobothnia | Pirkanmaa  Central Finland  South Karelia |
