# Supplementary material for: Contextual and mental health service factors in mental disorder-based disability pensioning in Finland – a regional comparison
Source: BMC Health Serv Res. 2021 Oct 11;21:1081. doi: 10.1186/s12913-021-07099-4 (PMC8507374; doi:10.1186/s12913-021-07099-4)
Supplement: Supplementary file 2 — Additional file 2: Appendix 2. Hospital district differences between all mental disorder–related disability pensions (DP), mood disorder (F30–39) DP and non–affective psychotic disorder (F20–29) DP in Finland, 2010–2015 by incidence rate ratio (IRR) and 95% confidence interval (95% CI). Crude model: Negative binomial regression model for hospital districts only. [file 12913_2021_7099_MOESM2_ESM.docx]

**Appendix 2.** Hospital district differences between all mental disorder–related disability pensions (DP), mood disorder (F30–39) DP and non–affective psychotic disorder (F20–29) DP in Finland, 2010–2015 by incidence rate ratio (IRR) and 95% confidence interval (95% CI).
Crude model: Negative binomial regression model for hospital districts only

|  | All mental disorder DP | | Mood disorder DP | | Non–affective psychotic disorder DP | |
| --- | --- | --- | --- | --- | --- | --- |
|  | IRR | 95% CI | IRR | 95% CI | IRR | 95% CI |
| National mean | 1.00 |  | 1.00 |  | 1.00 |  |
| Helsinki and Uusimaa (HUS) | 0.76 | 0.61 – 0.94 | 0.82 | 0.65 – 1.03 | 0.94 | 0.73 – 1.23 |
| Southwest Finland | 0.91 | 0.70 – 1.20 | 1.02 | 0.77 – 1.36 | 0.85 | 0.61 – 1.18 |
| Satakunta | 1.02 | 0.75 – 1.39 | 1.05 | 0.78 – 1.43 | 0.91 | 0.61 – 1.36 |
| Kanta–Häme | 0.77 | 0.62 – 0.97 | 0.75 | 0.60 – 0.94 | 0.81 | 0.57 – 1.14 |
| Päijät–Häme | 0.88 | 0.67 – 1.14 | 0.80 | 0.59 – 1.08 | 1.44 | 0.99 – 2.09 |
| Kymenlaakso | 1.14 | 0.85 – 1.53 | 1.18 | 0.87 – 1.59 | 1.18 | 0.79 – 1.76 |
| Pirkanmaa | 1.00 | 0.78 – 1.29 | 1.14 | 0.89 – 1.46 | 0.85 | 0.61 – 1.17 |
| Central Finland | 1.07 | 0.78 – 1.46 | 1.09 | 0.81 – 1.49 | 1.07 | 0.71 – 1.62 |
| North Savo | 1.14 | 0.89 – 1.47 | 1.29 | 1.02 – 1.64 | 1.05 | 0.72 – 1.53 |
| East Savo | 0.87 | 0.61 – 1.25 | 0.63 | 0.46 – 0.87 | 0.77 | 0.43 – 1.37 |
| South Savo | 1.02 | 0.77 – 1.34 | 1.00 | 0.74 – 1.35 | 1.10 | 0.75 – 1.63 |
| North Karelia | 0.90 | 0.70 – 1.15 | 0.86 | 0.68 – 1.07 | 1.29 | 0.88 – 1.89 |
| South Karelia | 1.02 | 0.77 – 1.35 | 1.05 | 0.80 – 1.38 | 1.10 | 0.71 – 1.72 |
| Vaasa | 0.71 | 0.55 – 0.91 | 0.67 | 0.51 – 0.88 | 0.66 | 0.40 – 1.09 |
| Länsi–Pohja | 1.09 | 0.76 – 1.56 | 1.10 | 0.78 – 1.55 | 0.80 | 0.43 – 1.47 |
| North Ostrobothnia | 1.27 | 0.95 – 1.70 | 1.39 | 1.04 – 1.87 | 1.26 | 0.88 – 1.80 |
| Central Ostrobothnia | 1.07 | 0.76 – 1.50 | 0.90 | 0.71 – 1.14 | 0.86 | 0.57 – 1.29 |
| South Ostrobothnia | 1.19 | 0.90 – 1.58 | 1.25 | 0.94 – 1.68 | 1.14 | 0.76 – 1.70 |
| Kainuu | 1.42 | 0.97 – 2.07 | 1.41 | 0.95 – 2.11 | 1.25 | 0.74 – 2.13 |
| Lapland | 1.04 | 0.75 – 1.45 | 1.07 | 0.76 – 1.49 | 1.08 | 0.65 – 1.78 |
| Nagelkerke Pseudo–R^2^ | 0.026 |  | 0.037 |  | 0.016 |  |
| AIC | 11957.71 |  | 10404.84 |  | 7050.523 |  |
| BIC | 12070.65 |  | 10517.77 |  | 7163.456 |  |
|  |  |  |  |  |  |  |
|  |  |  |  |  |  |  |
