# Supplementary figures and images for: Contextual and mental health service factors in mental disorder-based disability pensioning in Finland – a regional comparison
Source: BMC Health Serv Res. 2021 Oct 11;21:1081. doi: 10.1186/s12913-021-07099-4 (PMC8507374; doi:10.1186/s12913-021-07099-4)

**Appendix 3.** The correlation between the district-level factors


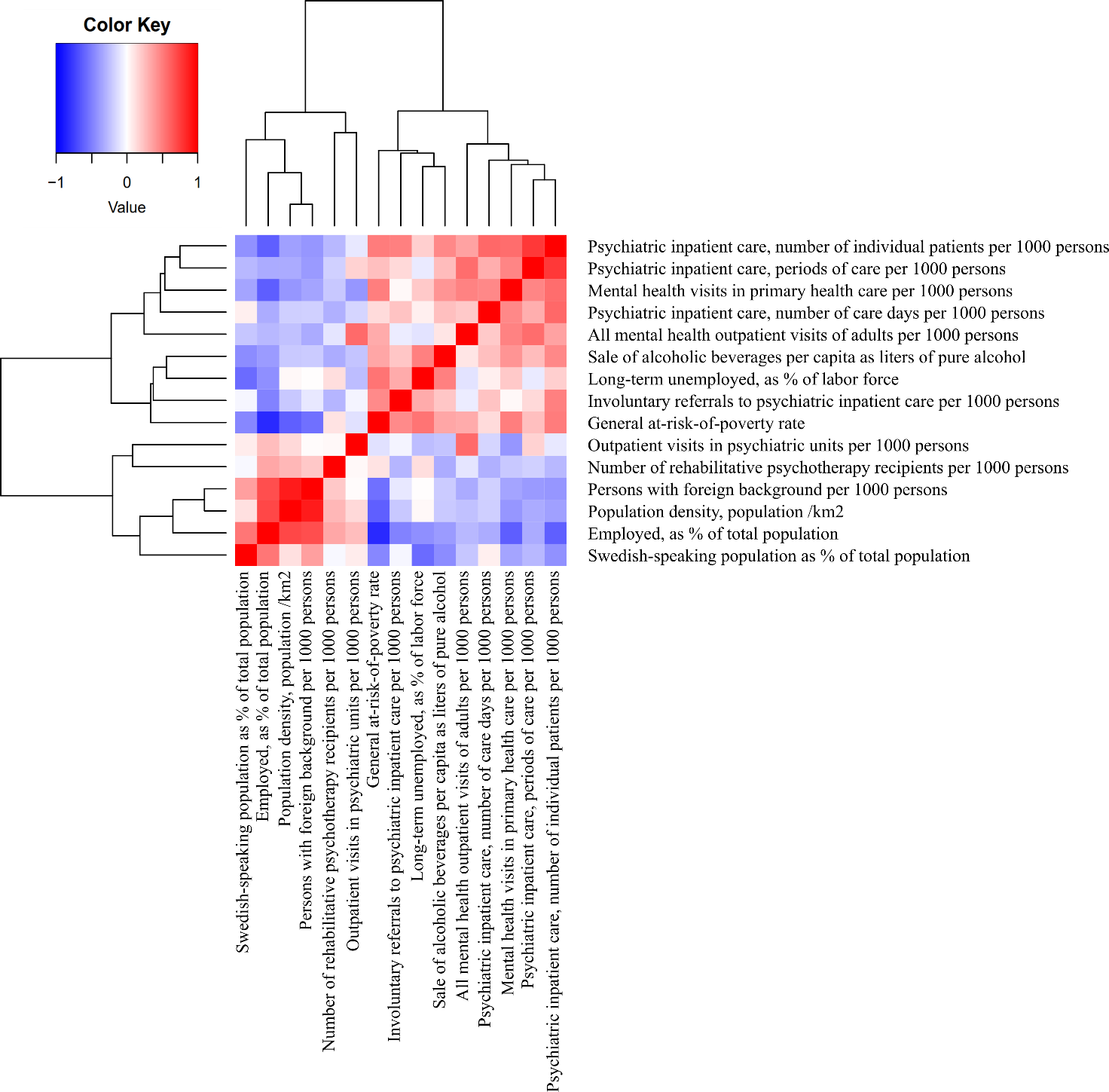

Supplement: Supplementary file 3 — Additional file 3: Appendix 3. The correlation between the district-level factors. [file 12913_2021_7099_MOESM3_ESM.docx]
