# Supplementary material for: Contextual and mental health service factors in mental disorder-based disability pensioning in Finland – a regional comparison
Source: BMC Health Serv Res. 2021 Oct 11;21:1081. doi: 10.1186/s12913-021-07099-4 (PMC8507374; doi:10.1186/s12913-021-07099-4)
Supplement: Supplementary file 4 — Additional file 4: Appendix 4. Associations of regional differences in contextual and mental health service factor for all mental disorder-related disability pensions (DP), mood disorder (F30–39) DP and non-affective psychotic disorder (F20–29) DP in Finland by incidence rate ratio (IRR) and 95% confidence interval (95% CI) (national mean as reference: 1.00) [file 12913_2021_7099_MOESM4_ESM.docx]

**Appendix 4.** Associations of regional differences in contextual and mental health service factor for all mental disorder-related disability pensions (DP), mood disorder (F30-39) DP and non-affective psychotic disorder (F20-29) DP in Finland by incidence rate ratio (IRR) and 95% confidence interval (95% CI) (national mean as reference: 1.00).

Crude model: Negative binomial regression model for contextual and mental health service factors only.

|  | All mental disorder DP | | Mood disorder DP | | Non-affective psychotic disorder DP | |
| --- | --- | --- | --- | --- | --- | --- |
|  | IRR | 95% CI | IRR | 95% CI | IRR | 95% CI |
| Swedish-speaking population as % of total population | p = 0.006 |  | p = 0.003 |  | p = 0.057 |  |
| Highest | 0.81 | 0.68 – 0.97 | 0.77 | 0.63 – 0.94 | 0.77 | 0.54 – 1.09 |
| Higher | 1.03 | 0.89 – 1.19 | 1.05 | 0.91 – 1.21 | 1.04 | 0.84 – 1.28 |
| Lower | 1.20 | 1.07 – 1.35 | 1.23 | 1.09 – 1.39 | 1.25 | 1.03 – 1.52 |
| Lowest | – |  | – |  | – |  |
| Persons with foreign background per 1000 persons | p = 0.004 |  | p = 0.044 |  | p = 0.077 |  |
| Highest | 0.84 | 0.75 – 0.94 | 0.86 | 0.76 – 0.98 | 0.84 | 0.72 – 0.98 |
| Higher | 1.06 | 0.95 – 1.19 | 1.06 | 0.95 – 1.19 | 1.13 | 0.97 – 1.32 |
| Lower | 1.12 | 1.03 – 1.23 | 1.09 | 0.99 – 1.20 | 1.05 | 0.93 – 1.19 |
| Lowest | – |  | – |  | – |  |
| General at-risk-of-poverty rate | p = 0.006 |  | p < 0.001 |  | p = 0.014 |  |
| Highest | 0.98 | 0.84 – 1.14 | 0.92 | 0.79 – 1.06 | 1.09 | 0.89 – 1.34 |
| Higher | 1.14 | 1.03 – 1.27 | 1.18 | 1.06 – 1.32 | 1.20 | 1.03 – 1.38 |
| Lower | 1.07 | 0.94 – 1.20 | 1.15 | 1.02 – 1.29 | 0.92 | 0.78 – 1.08 |
| Lowest | 0.84 | 0.74 – 0.95 | 0.81 | 0.72 – 0.90 | 0.84 | 0.71 – 0.98 |
| Employed, as % of total population | p = 0.002 |  | p = 0.004 |  | p = 0.081 |  |
| Highest | 0.77 | 0.67 – 0.88 | 0.78 | 0.68 – 0.91 | 0.84 | 0.69 – 1.03 |
| Higher | 1.04 | 0.93 – 1.17 | 1.07 | 0.95 – 1.19 | 0.94 | 0.81 – 1.10 |
| Lower | 1.12 | 1.01 – 1.25 | 1.16 | 1.04 – 1.29 | 1.16 | 1.01 – 1.34 |
| Lowest | 1.11 | 0.96 – 1.29 | 1.03 | 0.89 – 1.20 | 1.08 | 0.88 – 1.34 |
| Long-term unemployed, as % of labor force | p = 0.387 |  | p = 0.506 |  | p = 0.172 |  |
| Highest | 1.02 | 0.88 – 1.18 | 1.01 | 0.87 – 1.17 | 1.20 | 0.99 – 1.46 |
| Higher | 1.07 | 0.96 – 1.20 | 1.08 | 0.97 – 1.20 | 1.04 | 0.89 – 1.22 |
| Lower | 0.93 | 0.83 – 1.04 | 0.98 | 0.88 – 1.09 | 0.92 | 0.79 – 1.06 |
| Lowest | 0.98 | 0.85 – 1.14 | 0.94 | 0.81 – 1.08 | 0.87 | 0.71 – 1.07 |
| Sale of alcoholic beverages per capita, as liters of pure alcohol | p = 0.438 |  | p = 0.255 |  | p = 0.371 |  |
| Highest | 0.96 | 0.79 – 1.18 | 0.89 | 0.73 – 1.09 | 0.96 | 0.70 – 1.31 |
| Higher | 1.09 | 0.97 – 1.23 | 1.13 | 1.00 – 1.28 | 1.16 | 0.97 – 1.38 |
| Lower | 0.96 | 0.86 – 1.08 | 1.03 | 0.92 – 1.16 | 1.00 | 0.85 – 1.18 |
| Lowest | 0.99 | 0.84 – 1.15 | 0.96 | 0.83 – 1.12 | 0.90 | 0.72 – 1.12 |
| Population density, population/km² (proxy for the accessibility of treatment) | p = 0.014 |  | p = 0.098 |  | p = 0.720 |  |
| Highest | 0.83 | 0.71 – 0.96 | 0.86 | 0.73 – 1.01 | 0.95 | 0.79 – 1.14 |
| Higher | 1.04 | 0.93 – 1.16 | 1.04 | 0.93 – 1.17 | 1.00 | 0.87 – 1.15 |
| Lower | 1.16 | 1.05 – 1.28 | 1.12 | 1.01 – 1.24 | 1.06 | 0.93 – 1.20 |
| Lowest | – |  | – |  | – |  |
| All mental health outpatient visits of adults per 1000 persons | p = 0.002 |  | p < 0.001 |  | p = 0.817 |  |
| Highest | 1.21 | 1.00 – 1.45 | 1.24 | 1.04 – 1.49 | 1.08 | 0.83 – 1.39 |
| Higher | 0.97 | 0.87 – 1.08 | 0.93 | 0.84 – 1.03 | 0.95 | 0.82 – 1.11 |
| Lower | 0.79 | 0.70 – 0.89 | 0.76 | 0.67 – 0.85 | 0.94 | 0.79 – 1.11 |
| Lowest | 1.09 | 0.94 – 1.25 | 1.14 | 0.99 – 1.32 | 1.04 | 0.86 – 1.26 |
| Outpatient visits in psychiatric units per 1000 persons | p = 0.042 |  | p = 0.005 |  | p = 0.195 |  |
| Highest | 1.06 | 0.90 – 1.25 | 1.11 | 0.95 – 1.30 | 1.01 | 0.80 – 1.27 |
| Higher | 0.90 | 0.81 – 1.01 | 0.87 | 0.78 – 0.96 | 0.87 | 0.75 – 1.00 |
| Lower | 0.90 | 0.81 – 1.01 | 0.88 | 0.79 – 0.98 | 1.00 | 0.85 – 1.18 |
| Lowest | 1.16 | 1.01 – 1.34 | 1.18 | 1.02 – 1.36 | 1.14 | 0.94 – 1.38 |
| Mental health visits in primary health care per 1000 persons | p = 0.001 |  | p = 0.001 |  | p = 0.306 |  |
| Highest | 1.23 | 1.01 – 1.51 | 1.24 | 1.01 – 1.52 | 1.16 | 0.88 – 1.53 |
| Higher | 1.01 | 0.88 – 1.15 | 0.96 | 0.83 – 1.09 | 1.03 | 0.85 – 1.25 |
| Lower | 1.07 | 0.96 – 1.19 | 1.10 | 0.99 – 1.23 | 1.02 | 0.88 – 1.19 |
| Lowest | 0.75 | 0.65 – 0.87 | 0.77 | 0.66 – 0.90 | 0.82 | 0.66 – 1.01 |
| Number of rehabilitative psychotherapy recipients per 1000 persons | p = 0.958 |  | p = 0.708 |  | p = 0.612 |  |
| Highest | 0.97 | 0.84 – 1.12 | 1.00 | 0.87 – 1.15 | 1.13 | 0.91 – 1.39 |
| Higher | 1.01 | 0.85 – 1.18 | 1.02 | 0.88 – 1.19 | 0.97 | 0.77 – 1.23 |
| Lower | 0.97 | 0.85 – 1.11 | 0.93 | 0.82 – 1.06 | 1.09 | 0.89 – 1.34 |
| Lowest | 1.06 | 0.79 – 1.41 | 1.05 | 0.80 – 1.37 | 0.84 | 0.52 – 1.36 |
| Involuntary referrals to psychiatric inpatient care per 1000 persons | p = 0.722 |  | p = 0.380 |  | p = 0.199 |  |
| Highest | 1.01 | 0.89 – 1.14 | 0.98 | 0.86 – 1.11 | 1.18 | 1.00 – 1.40 |
| Higher | 0.95 | 0.83 – 1.09 | 0.94 | 0.83 – 1.07 | 0.86 | 0.71 – 1.04 |
| Lower | 1.06 | 0.95 – 1.18 | 1.09 | 0.98 – 1.21 | 1.01 | 0.88 – 1.17 |
| Lowest | 0.99 | 0.84 – 1.16 | 1.00 | 0.84 – 1.18 | 0.97 | 0.77 – 1.21 |
| Psychiatric inpatient care, periods of care per 1000 persons | p = 0.244 |  | p = 0.257 |  | p = 0.316 |  |
| Highest | 1.15 | 1.00 – 1.32 | 1.15 | 1.00 – 1.31 | 1.05 | 0.86 – 1.28 |
| Higher | 1.01 | 0.89 – 1.15 | 1.00 | 0.88 – 1.13 | 1.16 | 0.96 – 1.39 |
| Lower | 0.97 | 0.87 – 1.08 | 1.00 | 0.89 – 1.11 | 0.95 | 0.82 – 1.10 |
| Lowest | 0.89 | 0.74 – 1.06 | 0.88 | 0.73 – 1.05 | 0.87 | 0.67 – 1.12 |
| Psychiatric inpatient care, number of individual patients per 1000 persons | p = 0.164 |  | p = 0.134 |  | p = 0.282 |  |
| Highest | 1.05 | 0.93 – 1.19 | 1.00 | 0.88 – 1.13 | 1.16 | 0.98 – 1.38 |
| Higher | 1.07 | 0.96 – 1.21 | 1.05 | 0.94 – 1.18 | 1.02 | 0.86 – 1.20 |
| Lower | 1.04 | 0.93 – 1.16 | 1.10 | 0.99 – 1.23 | 0.95 | 0.82 – 1.10 |
| Lowest | 0.85 | 0.74 – 0.98 | 0.86 | 0.75 – 0.99 | 0.89 | 0.74 – 1.07 |
| Psychiatric inpatient care, number of care days per 1000 persons | p = 0.778 |  | p = 0.805 |  | p = 0.983 |  |
| Highest | 1.11 | 0.89 – 1.38 | 1.00 | 0.79 – 1.26 | 1.00 | 0.73 – 1.38 |
| Higher | 0.96 | 0.84 – 1.09 | 0.96 | 0.84 – 1.09 | 1.03 | 0.86 – 1.24 |
| Lower | 0.95 | 0.85 – 1.07 | 0.99 | 0.88 – 1.12 | 0.99 | 0.85 – 1.17 |
| Lowest | 0.99 | 0.85 – 1.15 | 1.06 | 0.91 – 1.23 | 0.97 | 0.79 – 1.20 |
